# Impact of different disinfection protocols on the bond strength of NeoMTA 2 bioceramic sealer used as a root canal apical plug (in vitro study)

**DOI:** 10.1038/s41405-024-00257-w

**Published:** 2024-09-23

**Authors:** Nada Omar, Nihal Refaat Kabel, Muhammad Abbass Masoud, Tamer M. Hamdy

**Affiliations:** 1 Restorative and Dental Materials Department, Oral and Dental Research Institute, National Research Centre (NRC), Giza, Dokki 12622 Egypt; 2 Pediatric Dentistry Department, Faculty of Dentistry, Misr University for Science and Technology (MUST), Cairo, Egypt; 3 Dental Biomaterials Department, Faculty of Dental Medicine, Boys, Al Azhar University, Cairo, Egypt

**Keywords:** Root canal treatment, Calcium-based cement

## Abstract

**Introduction:**

Treatment of an immature permanent tooth required a special disinfection protocol due to the presence of thin radicular walls, which are prone to fracture. Mineral Trioxide Aggregate (MTA) has been proposed as a root repair material for root canal treatment. The aim of this in vitro study was to compare the push-out bond strength of conventional White MTA cements and second generation NeoMTA 2 in imitated immature roots treated with different disinfection protocols, which are 5.25% sodium hypochlorite (NaOCl), followed by 17% ethylenediaminetetraacetic acid (EDTA), and NaOCl, followed by 20% etidronic acid (HEBP).

**Methods:**

The root canals of freshly extracted single-root teeth were manually prepared until 90 K-file to imitate immature roots. Roots were randomly divided into four groups (G) according to the disinfection protocol (*n* = 15 per group). where G1 (NaOCl + EDTA + White MTA) and G2 (NaOCl + EDTA + NeoMTA 2) While G3 (NaOCl + HEBP + White MTA) and G4 (NaOCl + HEBP + NeoMTA 2) All groups were activated with manual agitation. All specimens were incubated for 48 h. The apical third of each root was perpendicularly sectioned to attain a slice of 3 mm thickness. Push-out bond strength values were assessed using a two-way ANOVA and a Student’s *t* test.

**Results:**

G3 and G4 that were treated with HEPB showed higher significant push-out bond strength mean values than G1 and G2 treated with an EDTA chelating agent. Irrespective of the chelating agent used, it was found that both NeoMTA 2 and White MTA had no significant influence on push-out bond strength mean values (*p* ≤ *0.05*).

**Conclusion:**

The combined use of 5.25% NaOCl and 20% HEBP increased the push-out strength values of both NeoMTA 2 and White MTA, rendering them suitable to be used as an alternative chelating agent to EDTA.

## Background

Innovations in the root canal treatment comprise improvements in root canal filling materials, root canal irrigants, and instrumentation to achieve a suitable apical seal and convenient root canal treatment [1–5]. Treatment options for necrotic, immature permanent teeth include revascularization and apexification [6–8]. It has been demonstrated that revascularization of non-vital, immature roots is suggested in cases where deep caries or trauma has interrupted the normal root canal development [7, 8]; however, in other cases, it is not recommended and may lead to failure [9, 10]. In these situations, induction of apical closure utilizing the one-visit apexification technique by using a biocompatible, insoluble, and osteoconductive material, such as mineral trioxide aggregate (MTA), is becoming more reliable. where an apical plug is applied, filling the apical part of the immature root canals, which produce more favorable conditions for conventional root canal filling [10], and inducing an apical hard tissue matrix [11, 12].

MTA contains dicalcium and tricalcium silicate particles that set in a damp environment, forming calcium silicate hydrate. It is usually used in pulp capping, pulpotomy, root perforation repair, pulp regeneration, and root end filling materials [13–16]. However, its prolonged setting time, staining of the teeth, and difficulty in manipulation limit its use [17]. NeoMTA 2 is the second generation of NeoMTA, whose prototype was NeoMTA Plus [18]. It was developed to be a multipurpose root and pulp treatment material that is quicker to mix, whiter, higher radiopacity, and suitable for all procedures [18]. It is a fast-setting, bioactive, and non-staining material with easier manipulation to overcome the MTA drawbacks. It is resin-free for extreme MTA concentration and highest calcium and hydroxide ions release and maximum bioactivate potentiality [19]. Its unique gel properties ensure that the cement remains in place without being washed out. It doesn’t stain the teeth as it contains tantalum oxide as a radio-opacifier instead of bismuth oxide to overcome the discoloration potential [20–22]. It is composed of extremely fine, inorganic powder of tricalcium and dicalcium silicate with tantalum oxide and aluminum as a radiopacifying agent instead of bismuth oxide to overcome its well-known discoloration potential [18, 23].

Prior to starting the apexification procedures, disinfection of the canal is of prime importance because, in most cases, necrotic pulps are infected [24–26]. The primary phase of treatment is to disinfect the necrotic root canals to establish periapical healing [26]. It has been advocated that copious irrigation using sodium hypochlorite (NaOCl) and ethylenediaminetetraacetic acid (EDTA) be used for proper chemo-mechanical preparation, to control the microorganisms and their byproducts, to dissolve the necrotic tissue, and to remove the smear layer created during instrumentation [27–30]. Nevertheless, it was observed that the physical, chemical, and structural properties of dentin were altered when in contact with this combination of irrigants. NaOCl decreases the dentin microhardness, causing irreversible erosion of the dentin microstructure [31–33], denaturing the collagen components of the dentin surface and oxidizing the organic matrix. EDTA can change the ratio of organic and inorganic components of dentine, lowering the collagen matrix in mineralized tissues and thus altering its microstructure [34–36]. Considering these facts of clinical occurrences, especially in immature permanent teeth, they may develop a more brittle and less resistant tooth structure substrate. Subsequently, the endo-treated teeth will be more susceptible to crown or root fractures [37]. Etidronic acid, also referred to as HEBP (1-hydroxyethylidene-1,1-bisphosphonate) (BP), is a weak, biocompatible chelating solution that has an adequate calcium chelation capacity, is reportedly less abrasive to root dentine than EDTA, and could be utilized in conjunction with NaOCl [38–41]. It has the ability to chelate metallic ions. It has been suggested as a potential alternative to EDTA [42]. The concentration of HEBP is a crucial factor for effective removal of calcium from the root canal as the lower concentrations are less efficient [43]. Although etidronic acid were tested as an irrigant solutions in a previous study, the effect of compositional alterations of NeoMTA 2 in combination with 5.25% NaOCl and 20% HEBP irrigation protocol on the push-out bond strength has not been reported.

Hence, the aim of this in vitro study was to compare the push-out bond strength of the conventional White MTA cements and the second generation NeoMTA 2 as root end fillings in simulated immature permanent teeth treated with different disinfection protocols, which are 5.25% NaOCl, followed by 17% EDTA, and 5.25% NaOCl, followed by 20% HEBP. The null hypothesis was that there was no significant difference when using 5.25% NaOCl, followed by 17% EDTA, and 5.25% NaOCl, followed by 20% HEBP, among the following two root-end filling materials:

## Methods

### Sample collection

The present experimental study was approved by the Medical Research Ethical Committee (MREC) of the National Research Centre (NRC), Cairo, Egypt (Reference number: 3587062022). All methods were performed in accordance with the Declaration of Helsinki. Forty freshly extracted permanent, straight, single-rooted human teeth were gathered from the oral surgery dental clinic in the National Research Centre, Cairo. Extractions were performed with consent. Teeth were inspected under stereomicroscopy (×10) to eliminate roots with cracks, fractures, and caries. Also, they were radiographed in the mesiodistal and buccolingual aspects to detect any resorption. Exclusion of teeth with decay, cracks, or fractures Teeth were scaled to remove any calcified deposits. Organic tissues and any remaining soft tissue were removed by immersion of the teeth in 5.25% NaOCl for 10 min. Finally, they were stored in distilled water until use.

### Specimen size determination

Sample size was determined using sample size calculator software program (G. power 3.19.2) based on research published by Buldur et al., and Shetty et al. [44, 45]. Sample size calculation was based on 95% confidence interval and power of 90% with α error of 5%. The minimum sample size estimated for this study was 15 samples in each group.

### Samples preparation

The coronal segments of all samples were sectioned by sectioning disc mounted on a low speed handpiece along with water coolant to standardize the teeth lengths at 15 mm. Mechanical preparation was done using ProTaper Next system (files X1- X3) (PTN; Dentsply Maillefer, Ballaigues, Switzerland). The canals were irrigated with 5 mL of freshly prepared 5.25% NaOCl solution, followed by a rinse with 5 ml distilled water.

### Root-end preparation and plug condensation

All roots were resected perpendicular to the root’s long axis by a sectioning disc, and 3 mm were removed apically. A balanced force technique was used for apical enlargement until file K-90 (Dentsply/Maillefer, Ballaigues, Switzerland).

The simulated immature roots were arbitrarily divided into four experimental groups (*n* = 15 per group) according to the irrigation protocol and apical plug material as follows:

Group 1 (G1): Samples were irrigated by utilizing 5 ml NaOCl 5.25% (Sigma-Aldrich, Inc., St. Louis, MO, USA), followed by 5 ml 17% EDTA (Sigma-Aldrich, Inc., St. Louis, MO, USA), activated with manual agitation for 5 min., followed by 5 ml distilled water as a final rinse. Canals were slightly dried with paper points and a 5 mm apical plug using White MTA (Pro Root MTA, Dentsply Tulsa Dental, Tulsa, OK, USA) that was prepared according to the manufacturer’s recommendations and incrementally placed in orthograde direction using the MAP system (Roydent, Johnson City, TN, USA) and further compacted with a pre-fitted plugger.

Group 2 (G2): irrigation using 5 ml of 5.25% NaOCl followed by 5 ml of 17% EDTA. Canals were slightly dried using paper points, and an apical plug using Neo MTA 2 (NuSmile Avalon Biomed, Bradenton, FL, USA) was prepared the same way in G1.

Group 3 (G3): irrigation using 5 ml of 5.25% NaOCl along with 5 ml of 20% HEBP (Cublen K8514 GR; Zschimmer & Schwarz, Mohsdorf, Germany) activated manually for 5 min. A 5 mm apical plug using white MTA. Prepared according to the respective manufacturer’s recommendations and incrementally placed in an orthograde direction.

Group 4 (G4): irrigation using 5 ml of 5.25% NaOCl along with 5 ml of EDTA activated manually for 5 min. A 5 mm apical plug using Neo MTA 2. It was prepared and placed as before.

All specimens were labeled and stored in an incubator (CBM, S.r.l. Medical Equipment, 2431/V, Cremona, Italy) at 100% humidity at 37 °C for 48 h to ensure the complete hardening of the tested cements [46–48]. White MTA and NeoMTA 2, regarding push-out bond strength. The composition of the two root-end filling materials examined in the current study are represented in Table 1.

**Table 1:** The composition of the root-end filling materials utilized in the study.

Root-end filling materials	Chemical composition^a^	Manufacturers
White MTA	Powder: SiO_2_, K_2_O, Al_2_O_3_, Na_2_O, Fe_2_O_3_, SO_3_, BI_2_O_3_, MgO, CaO insoluble residues and crystallized silicon Liquid: Distilled water	Pro Root MTA, Dentsply Tulsa Dental, Tulsa, OK, USA
NeoMTA 2	Powder: Ca_3_SiO_5_), Ca_2_SiO_4_), Ta_2_O_5_, and minor amounts of CaSO_4_ and Ca_3_Al_2_O_6_ Liquid: Distilled water and proprietary polymers	NuSmile Avalon Biomed, Bradenton, FL, USA

^a^The chemical composition taken from the relevant Material Safety Data Sheets (MSDS).

### Push-out test procedure

The roots apical thirds were sectioned horizontally, perpendicular to their long axis, with a water-cooled precision saw, obtaining a 3 mm (0.1) section in thickness. Sections were gauged by a digital caliper (Pachymeter, Electronic Digital Instruments, China). Each specimen was labeled and pictured coronally and apically using a stereomicroscope (65x) (SZ-PT; Olympus, Tokyo, Japan). A scale was conducted by matching up a ruler of a recognized length using the “Set Scale” tool of the image analysis software (Image J; NIH, Bethesda, MD, USA). The diameter of the filling was measured, and subsequently, the radius was calculated. Every section was mounted in a custom-made loading fixture (a metal block with a circular cavity in the middle). The hole for specimen housing had a central cavity to ease the movement of extruded cement material. A computer-controlled compressive load with a crosshead speed of 1 mm/min on a testing machine (Model 3345; Instron Industrial Products, Norwood, MA, USA) was applied to each specimen.

A load was applied to the specimens’ radicular parts by a plunger of 0.75 mm in diameter. The tip of the plunger was positioned only touching the cement part, avoiding the surrounding dentin, in an apical-coronal direction to avoid any obstruction of the cement movement towards the wider diameter. This guaranteed that during the loading process, the overlaying dentin was efficiently supported.

The maximum load failure (in Newton) was recorded and then converted into MPa. The bond strength was calculated by recording the maximum load and dividing it by the computed surface area, calculated by the following formula [40, 49]:

$$[{{\rm{A}}}=\left(\right. 3.14 * {{\rm{H}}}* ({{{\rm{r}}}}^{1}+{{{\rm{r}}}}^{2})]$$

Where; r^1^: apical radius, r^2^: coronal one, h: the thickness of the sample in mm.

**Fig. 1:**
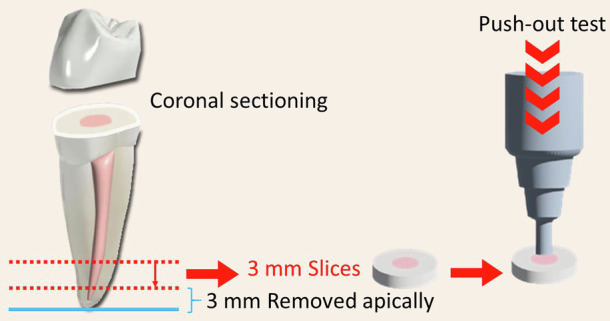
Diagrammatic illustration of the specimen’s preparation. It provides  the sectioning of the crown and the apical parts.

The push-out bond strength was determined for each root specimen. Failure was demonstrated by the displacement of the cement out of the canal lumen. The sudden drop in the load-deflection curve confirms bond failure, as recorded by Blue-hill computer software (62.01, version 2.0, NY, USA). Figure 1 represent diagrammatic illustration of the specimen’s preparation.

### Statistical analysis

Statistical Package for Social Sciences (SPSS, IBM, Chicago, USA) 16.0 statistical software was used to conduct the statistical study. The normality test carried out by Kolmogrov–Smirnov and Shapiro–Wilk tests; the data exhibited a normal distribution. After utilizing various irrigants, a two-way ANOVA and a Tukey test were used to compare the mean push-out bond strength values (MPa) for the various root-end filling materials. The significance level was set at *P* ≤ 0.05.

## Results

The mean values and standard deviation of the push-out bond strength (MPa) as function of chelating agent subgroup and different root-end filling materials were outlined in Table 2.

**Table 2:** Push-out bond strength (Mean and Standard Deviation) regarding the material groups after using various irrigation protocol.

Root end fillings	Irrigation protocol		*Statistics*
	EDTA chelating agent	HEBP chelating agent	*P value*
White MTA	4.327 ± 1.016	5.203 ± 0.395	0.04*
NEO MTA 2	4.229 ± 1.212	5.331 ± 1.039	0.05*
*P value*	0.85	0.73	

^*^significant (*p* ≤ 0.05).

As regards chelating agents, HEBP showed higher significant push-out bond strength mean values (G3 and G4) than EDTA when used with either White MTA or NeoMTA 2 (*P* = 0.04 and 0.05, respectively) (G1 and G2).

Comparing the two root-end filling materials (White MTA and NeoMTA 2), when EDTA chelating agent was used as an irrigation protocol, there was no significant difference in their bond strength mean values (*P* = 0.85). Similarly, the HEBP chelating agent resulted in an insignificant difference between the bond strength mean values of White MTA and NeoMTA 2 (*P* = 0.73).

Moreover, two-way ANOVA showed that the interaction of variables (Root canal filling type and chelating agent protocol) was not significant (*P* = 0.24). While, the effect of chelating agent protocol separately was significant (*P* = 0.0001), contrary to the effect of type of root canal filling separately was insignificant (*P* = 0.87), as represented in Table 3.

**Table 3:** Interaction of variables and effect of each factor.

Variables	*P* value
Root canal filling type	0.87
Chelating agent	0.0001*
Root canal filling type X Chelating agent protocol	0.24

*significant (*p* ≤ 0.05)

## Discussion

Achievement of a perfect seal at the apex of immature necrotic teeth and protection of the remaining tooth structure of the immature tooth using a bioinert filling material after efficient root canal debridement are the most important factors for the success of its treatment [50, 51].

The management of immature roots is accompanied by many challenges. Its difficulty in debridement of the root canal due to thin roots at risk of fracture and the absence of an apical stop makes root canal filling difficult [52]. These obstacles can be controlled by enhancing the synthesis of a hard tissue barrier at the root end and augmenting the root against fracture by the apexification technique [53, 54]. Several dental materials were used for the formation of the apical barrier, such as calcium hydroxide, freeze-dried dentin, freeze-dried cortical bone, dentin shavings, resorbable ceramic, bone morphogenic protein, MTA, Biodentine, and the recently introduced NeoMTA 2 cement [27, 55].

On the other hand, although root canal irrigation is an efficient way for its debridement [56], it was nevertheless revealed that several chemical irrigants induce alterations in the dentine walls [28]. The most common and widely applied irrigation protocol includes the use of NaOCl followed by a final flush with EDTA [27, 30, 42]. EDTA is efficient in the removal of the smear layer due to its chelating effect. Though its erosive effect hinders the mechanical characteristics of root dentin by modifying its calcium to phosphorous ratio [57, 58], it causes reduction in dentine microhardness, increasing solubility and permeability properties [59, 60].

An alternative combination of NaOCl and a weak chelating agent such as etidronic acid (HEBP) has been advocated because it maintains the properties of both individual solutions and decreases the deleterious effect of EDTA on root canal dentine [40, 41, 59]. Yadav et al. was reported that the using of concentration of 18% HEBP was more effective than concentration of 9% HEBP in removing calcium from the root canal due to the higher concentration. [43].

The adhesion of root-end filling cements to the dentinal walls is one of the significant essentials for success. providing a good root end seal filling material-dentin interface, increasing the ability to pack the root canal filling in the immature roots, and maintaining the integrity of the remaining short, underdeveloped roots [61]. Nevertheless, the kind of material used for apexification can directly affect the quality of its bonding to the dentin [62]. In addition to the chemicals used to debride the necrotic, immature, weak permanent teeth [63]. A root-end filling material should be stable against displacement and dislodging pressures. Push-out bond strength testing is an efficient and reliable way to determine how well a material fits into the surrounding root dentin and how well root-end filling materials resist dislodgement to demonstrate their efficacy [64–66]. The push-out strength test was conducted after 48 h of material mixing, as it was reported as the most appropriate time to ensure material hardening and the most crucial time to test the bond strength [46–48, 67–69].

Consequently, the current study was outlined to evaluate and compare the push-out bond strength of two calcium silicate-based cements used as root end filling materials in simulated immature roots with different irrigation protocols. According to the above findings, the null hypothesis was rejected, as regardless of the apical plug materials used, the type of chelating agent used in disinfection of immature root canals made a significant difference in the push-out bond strength.

Regardless of the apical plug material used, it was observed that when the root canals were treated with 20% HEBP in G3 and G4, they showed greater push-out bond strength mean values than in G1 and G2, where 17% EDTA was used when used following 5.25% NaOCl. This can be referred to as the minimal action of HEBP on dentine physical properties, interfering minimally with the microhardness and roughness of the dentinal walls. Also, it was mentioned in previous studies that HEBP caused the least change in the ability of NaOCl to breakdown organic matter and had the least erosive effect on dentine [70, 71]. Therefore, it could be more suitable for disinfection of the canals and dissolving of necrotic tissue in combination with NaOCl without further weakening of the root canal dentine. Furthermore, other studies reported MTA-dentin bond failures after irrigation with NaOCl and EDTA [72–74]. This may be related to the erosive and dissolving effects of the EDTA chelating agent, rendering dentine weaker for bonding with MTA [75]. This finding is in accordance with Barrio et al., who showed that irrigation with NaOCl and HEBP after repairing a root canal perforation with calcium silicate-based cements has no detrimental effect on the bond strength of these materials [5].

The combined use of 5.25% NaOCl and 20% HEBP increased the push-out strength values of both NeoMTA 2 and White MTA, rendering them suitable to be used as an alternative chelating agent to EDTA.

The findings of the current study suggest that the treatment of White MTA or Neo MTA 2 with 5.25% NaOCl followed by 20% HEBP solutions provides a stronger bond to the root canal dentine than the treatment with chelating agent to EDTA. Further research is needed to confirm the clinical usage of the suggested irrigation protocol with root end filling materials. Moreover, it is suggested that the irrigant protocol is a significant variable affecting the push-out bond strength more than the type of MTA.

## Conclusions

Within the limitations of the current in vitro study, it could be concluded that the combined use of 5.25% NaOCl and 20% HEBP increased the push-out strength values of both White MTA, and NeoMTA 2 rendering them suitable to be used as an alternative chelating agent to EDTA. Moreover, the employed chelating agent within the disinfection protocol had a great influence on the bond strength between dentin and apical plug materials.

## Data Availability

The data that support the findings of this study are available from the corresponding author upon reasonable request.
